# E-cadherin might be a stage-dependent modulator in aggressiveness in pancreatic cancer cells

**DOI:** 10.3906/biy-1912-60

**Published:** 2020-10-13

**Authors:** Esra AYDEMİR ÇOBAN, Didem TECİMEL, Ezgi KAŞIKCI, Ömer Faruk BAYRAK, Fikrettin ŞAHİN

**Affiliations:** 1 Department of Genetics and Bioengineering, Engineering Faculty, Yeditepe University, İstanbul Turkey; 2 Department of Medical Genetics, Faculty of Medicine, Yeditepe University, Yeditepe University Hospital, İstanbul Turkey; 3 Department of Microbiology & Immunology, Albert Einstein College of Medicine, New York, NY USA

**Keywords:** Panc-1, AsPC1, E-cadherin, CRISPR/dCas9 activation, pancreas cancer

## Abstract

Pancreatic ductal adenocarcinoma (PDAC) pathology is known for its uncontrollable progress due to highly invasive characteristics and refractory behavior against existing chemotherapies. The aberrant expression of CDH1 (expresses the protein E-cadherin) is associated with increased overall survival in various cancers, however, E-cadherin expression in PDAC progression has remained elusive. We investigated the impact of exogenously elevated E-cadherin levels on the tumorigenicity of transduced low grade and metastatic PDAC cell lines, Panc-1 and AsPC-1, respectively. Constitutive expression of E-cadherin promoted a more hybrid E/M state in AsPC-1 cells, while it was associated with the acquisition of a more epithelial-like state in Panc1 cells. Our study suggests that E-cadherin may play differential roles in determining the metastatic characteristics of primary and metastatic pancreatic cancer cells.

## 1. Introduction

In terms of availability of effective therapies, pancreatic ductal adenocarcinoma (PDAC) remains a challenging tumor both due to the intracorporeal location of the pancreas and strong resistance that rapidly develops to chemotherapy (Swayden et al., 2018). Most PDAC cases are diagnosed at late stages where the tumor has already metastasized and/or surgical intervention is no longer possible (Moletta et al., 2019). Pancreatic cancer owes its aggressive biology to its cancer stem cell (CSC)-like characteristics, whereby pancreatic cancer cells display abilities of self-renewal, efflux of drugs, and retention of migration properties. Maintenance of CSCs is enabled in a process called epithelial to mesenchymal transition (EMT), in which cells undergo morphological changes from an epithelial to a mesenchymal phenotype accompanied by the concomitant induction in the expression of those transcription factors related to EMT (Bhagwandin and Shay, 2009).

EMT-promoting signaling pathways are associated with malignant transformation. A clear-cut of losing all epithelial characteristics while gaining mesenchymal ones stays as theoretical because the cells remain more like in an intermediate phenotypic spectrum (Zhang and Weinberg, 2018). When cancer cells acquire mesenchymal traits and simultaneously express epithelial features, this interphase state is described as hybrid E/M (Kroger et al., 2019). The *CDH1* gene (which encodes the E-cadherin protein and will be referred to as such in the remainder of the manuscript) is the main gene that mediates the epithelial characteristics via binding to the anchoring catenin proteins. Through bridging E-cadherin with the actin cytoskeleton, catenins contribute to E-cadherin’s key role in conferring the adherent properties of the epithelial cells. However, the function of E-cadherin in cancer remains elusive due to the existence of opposing results reported by different studies. As loss of cell-to-cell adhesion and acquisition of mesenchymal markers is associated with the migratory capacity of cells, E-cadherin has been considered as a tumor suppressor gene for years. Chao et al. suggested that E-cadherin reexpression provides a survival advantage to the malignant cells and exerts a protecting effect on micrometastases (Chao et al., 2010) that lowers the cytotoxicity of chemotherapy in prostate cancer. Interestingly, E-cadherin is expressed in most carcinomas, including metastatic ones (Petrova et al., 2016). Suppression of E-cadherin expression is associated with a diminished rate of proliferation and viability (Song et al., 2019). Based on these facts, the role of E-cadherin in cancer biogenesis needs to be described more precisely. Hence, the goal of this study was to investigate the role of E-cadherin expression in a primary and metastatic pancreatic cancer cell line. 

## 2. Material and methods

### 2.1. Cell cultures 

The parental Panc-1 (ATCC®, USA) cell line was cultured with Dulbecco’s Modified Eagle’s Medium, High Glucose (GIBCO, USA); the parental AsPC-1 (ATCC®, USA) cell line was cultured with RPMI 1640 Medium (GIBCO, USA). Both media formulations were supplemented with 10% fetal bovine serum (FBS) (GIBCO, USA) and 1% penicillin/streptomycin/amphotericin (PSA) (GIBCO, USA). The media were changed every two days and the cells were incubated in a humidified chamber at 37 °C in 5% CO2. 

### 2.2. Viral particles and gene activation 

Panc-1 and AsPC-1 cells were transduced with lentiviral activation particles of CDH1 (Santa Cruz Biotechnology, Inc., Santa Cruz, CA, USA) and control lentiviral activation particles (Santa Cruz Biotechnology, Inc.) that lack any protein-coding gene sequences as instructed by the manufacturer (Ran et al., 2013). CDH1-activated cells were named EPanc-1 and EAsPC-1 and control cells were named cPanc-1 and cAsPC-1. 

### 2.3. Immunocytochemistry analysis

E-cadherin-activated and control Panc-1 and AsPC-1 cells were seeded on an 8-well chamber slide at a density of 104 cells/well. Briefly, the cells were washed with phosphate-buffered saline (PBS) and fixed with 4% paraformaldehyde (P6148, Sigma-Aldrich Corp., St. Louis, MO, USA) for 20 min at room temperature. Cells were then washed with PBS and permeabilized with 0.1% Triton-X 100 (X100, Sigma-Aldrich Corp.) for 20 min at room temperature. This procedure was followed by blocking with 10% goat serum (GS, 31872, Thermo Fisher Scientific Inc., Waltham, MA, USA) for 1 h and conjugating E- cadherin, vimentin, and Snail antibodies (Human EMT-3 Color Immunocytochemistry Kit, Cat. No. SC026, R&D Systems, Inc., Minneapolis, MN, USA) at room temperature for 3 h at a concentration 1:50 in PBS. DAPI (5 µg/mL) was used for nuclear staining and slides were viewed under a confocal microscope. Images were taken at 20 × magnification.

### 2.4. Western blot analysis 

Cellular pellets were collected and lysed using the radio-immunoprecipitation assay (RIPA) buffer (Sigma-Aldrich Corp., USA) supplemented with protease and phosphatase inhibitor cocktail and probed against primary antibodies including E-cadherin, Snail, Slug, N-cadherin, Zeb-1, vimentin, and β-catenin (antibodies included in EMT sampler kit, Cell Signaling Technology, Inc., Danvers, MA, USA); Nf-κB p65 (Cell Signaling Technology, Inc.); phospho-Akt (Ser 473) (Cell Signaling Technology, USA); Met, CD44, c-MYC (antibodies included in Wnt/β-catenin activated targets sampler kit, Cell Signaling Technology, Inc.); Klf4 (R&D Systems, Inc.); Nanog (R&D Systems, Inc.), GAPDH (Cell Signaling Technology, Inc.).

### 2.5. Migration and invasion assay 

CDH1-activated Panc-1 and AsPC-1 cells were seeded onto 6-well plates at 90% confluence and the scratch assay was performed according to the manufacturer’s instructions (Liang et al., 2007). As for the invasion assay, EAsPC-1 cells were trypsinized and cultured in invasion Boyden chambers (Cell Biolabs, Inc., San Diego, CA, USA) according to the manufacturer’s protocol (Quinn et al., 2017).

### 2.6. Sphere formation assay 

CDH1-transduced Panc-1 and AsPC-1 cells were cultured in nonadherent cell culture plates and treated with sphere formation media (SFM) consisting of 2% B27, 1% insulin-transferin-selenium (ITS) (GIBCO, USA), 1% PSA, 20 ng/mL bFGF (GIBCO, USA), 20 ng/mL rhEGF (GIBCO, USA), and 0.4% BSA (GIBCO, USA). Cells were incubated, with replenishing SFM every 2 days, until spheres with a diameter ranging from 40 µM (smallest) to 128 µM were observed and evaluated.

### 2.7. Colony formation assay 

CDH1-activated Panc-1 and AsPC-1 cells were seeded onto 6-well plates at densities of 250, 500, and 1000 cells/well. Cells were incubated for two weeks, stained with 0.5% crystal violet solution (Sigma-Aldrich Corp.), and average colony numbers were calculated.

### 2.8. Statistical analysis

Statistical analysis was done with GraphPad Prism 5 (GraphPad Software, La Jolla, CA, USA) and comparisons were made by using two-way analysis of variance (ANOVA) and Bonferroni’s test as a posttest and unpaired t-test, where applicable. The value of P < 0.05 was considered statistically signiﬁcant.

## 3. Results

### 3.1. Immunocytochemistry analysis

**Figure 1:**
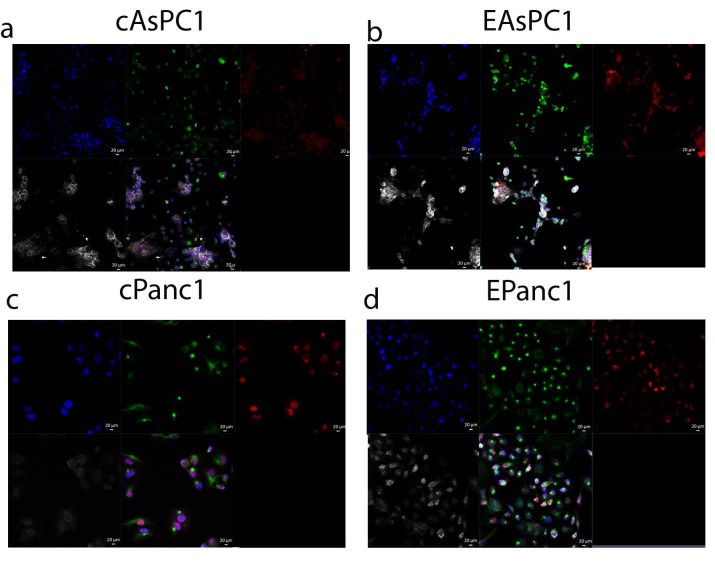
(a–d) Triple immunocytochemistry staining of EMT markers; Snail(red)/vimentin(green)/E-cadherin(white) and cell nucleus stained with DAPI.

The levels of EMT proteins upon E-cadherin activation were evaluated in both cell lines. According to this, Snail (red signal, Figures 1a–1d), which is an important repressor of E-cadherin, was upregulated in both cell lines suggesting a hybrid state of EMT/MET. 

### 3.1. Confirmation of E-cadherin activation and expression patterns of EMT

**Figure 2:**
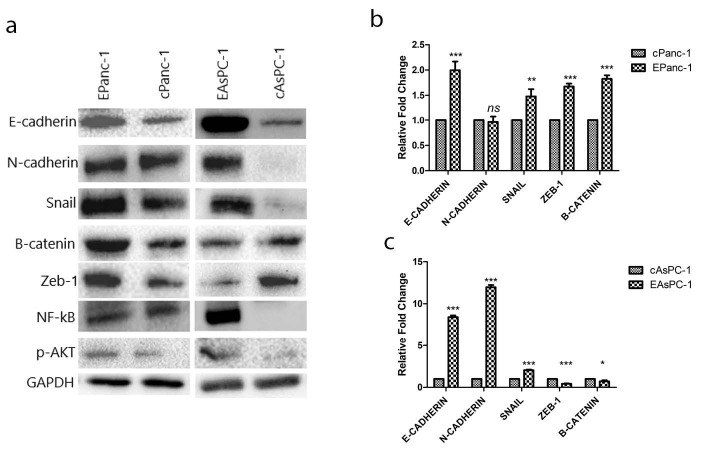
(a) Representative western blot images for both cell lines. (b) Relative bar graphs for the changes in EMT and EMTrelated protein expressions in EPanc-1 and cPanc-1 cells. (c) Relative bar graphs for the changes in EMT and EMT-related protein expressions in EAsPC-1 and cAsPC-1. Mean ± SD; *P < 0.05, **P < 0.01, and ***P < 0.001, vs. the control group.

Figure 2a shows that the initial protein level of E-cadherin is lower in Panc-1 cells when compared to the AsPC-1 cells. The overexpression of E-cadherin and the fold changes for other EMT-related markers upon it are presented in Figure 2a. EMT markers, including ZEB-1 and β-catenin, were upregulated in EPanc-1(Figure 2b) and downregulated in EAsPC-1 (Figure 2c). A significant increase in N-cadherin for EAsPC-1 cells was also recorded (Figure 2c). 

### 3.2. Migration and invasion assay 

**Figure 3:**
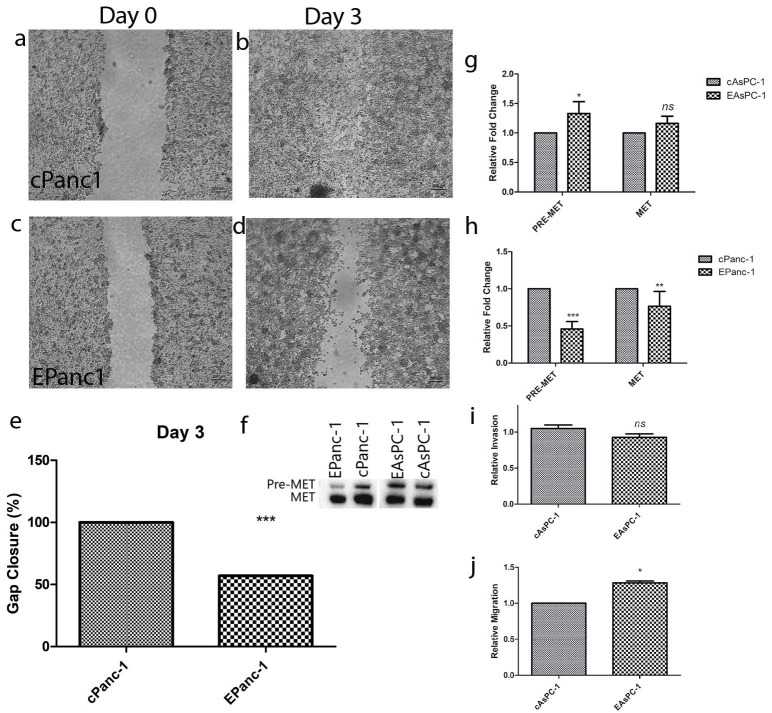
(a–e) Forced activation of E-cadherin-inhibited EPanc-1 cell migration by gap closure assay. (f–h) ThThe changes in protein expression levels of pre-Met and Met. (i–j) ThThe invasion and migration capacity of EAsPC-1 cells are analyzed. Mean ± SD; *P < 0.05, **P < 0.01, and ***P < 0.001, vs. the control group.

The migration properties of EPanc-1 cells were analyzed with the scratch assay (Figures 3a–3d). EPanc-1 cells closed 57% of the scratched area within 4 days, while cPanc-1 cells migrated at a faster rate, closing the gap completely within 4 days (Figure 3e). Due to the inconvenience of AsPC-1 cells, which float freely in the cell culture environment (Chen et al., 1982), we were unable to measure the possible changes in migration using the scratch assay. Therefore, only protein quantification data for the metastatic marker, preMet/Met, was determined as a read-out for alterations in migration (Figure 3f). Also, pre-Met levels were upregulated in EAsPC-1 cells in contrast to those in EPanc-1 cells (Figure 3f). According to this, pre-Met was slightly (<1.5-fold) upregulated in EAsPC-1 cells (Figure 3g), whereas it was downregulated in EPanc-1 cells (Figure 3h). Since it was not feasible to apply the scratch assay to the poorly adherent AsPC-1 cells, the invasion and migration assays were employed to evaluate their migratory capacity. Based on our findings, the invasive potential of AsPC-1 cells remained the same as before the exogenous increase in the E-cadherin expression (~1 fold) (Figure 3i), while the migration of the EAsPC-1 cells was slightly increased (<1.5-fold) compared to cAsPC-1 cells (Figure 3j).

### 3.3. Sphere and colony formation assay 

**Figure 4:**
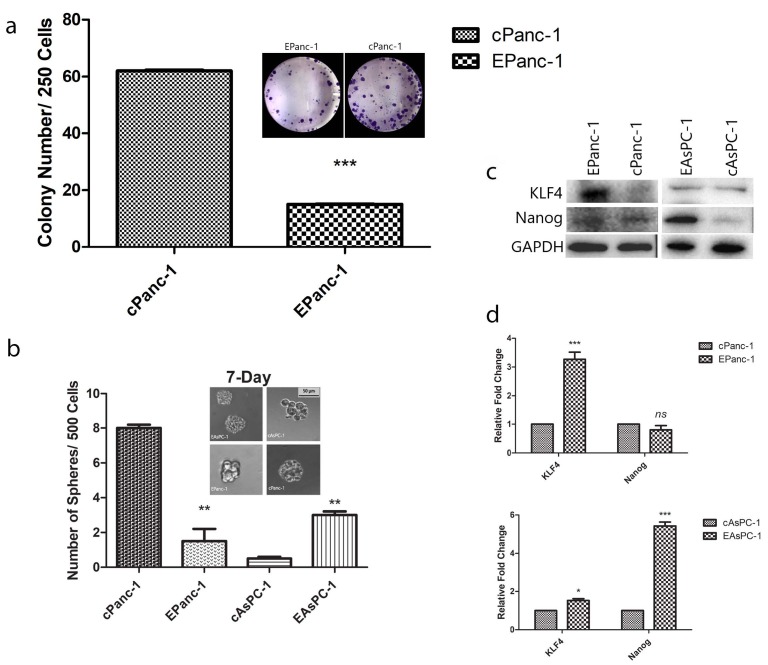
(a) Forced activation of E-cadherin-inhibited EPanc-1 cell colony formation assessed with the colony formation assay. A representative image and bar graph for colony numbers from 3 independent experiments are shown. (b) Inhibition in the sphere-forming capacity of EPanc-1 cells while triggering the sphere formation in EAsPC-1 cells. (c–d) Klf4 and Nanog expressions are shown. Mean ± SD; *P < 0.05, **P < 0.01, and ***P < 0.001, vs. the control group.

In our study, the formation of colonies and spheres (ranging between 40 to 140 µm in diameter) was 4 times less in EPanc-1 cells than in cPanc-1 cells (Figures 4a–4b). Both cAsPC-1 and EAsPC-1 cells were unable to form colonies (data not shown); rather, the cAsPC-1 cells formed a botryoid (Figure 4b). On the other hand, EAsPC-1 cells were able to form a regular spheroid form within the given diameter range (Figure 4b). Increased levels of the Krüppel-like factor (KLF4) (Figure 4c) were measured in EPanc-1 cells, while a similar increase in EAsPC-1 cells was absent. Nanog levels (Figure 4c) were elevated in EAsPC-1 cells, whereas it was not elevated in EPanc-1 cells.

## 4. Discussion

While we do not wish to generalize from our limited set of experiments, we would consider evaluating the effect of overexpression of E-cadherin on the aggressiveness of differentially behaving pancreatic cancer cell lines. Our findings show that overexpression of *CDH-1* in both primary and metastatic pancreatic cancer cell lines was not sufficient to attain complete epithelial characteristics. Rather, both cell lines remained in interphase in the progression from a mesenchymal to an epithelial state where coexpression of both epithelial and mesenchymal markers was evident, in line with the hybrid EMT/MET model (Jolly et al., 2015; Smigiel et al., 2018). Transition states in both directions, either from epithelial-to-mesenchymal or mesenchymal-to-epithelial are reversible processes in nature (Chao et al., 2010). According to this model, being in the transit hybrid E/M state has a more aggressive phenotype than retaining either the E or M state properties that display diminished tumorigenicity (Kroger et al., 2019).

β-catenin expression is essential for cadherin-mediated cell-to-cell adhesion as it is the anchoring protein connecting the actin cytoskeleton and cadherin cytoplasmic domain (Heuberger and Birchmeier, 2010). Seidel et al. demonstrated that when E-cadherin and N-cadherin are stably expressed in an E-cadherin and N-cadherin deficient pancreatic cancer cell line MIA-PaCa-2, only restoration of E-cadherin expression promoted an upregulation in β-catenin levels (Rosivatz et al., 2004). Results from our study confirmed this positive correlation between E-cadherin and β-catenin expression in the EPanc-1 cells. However, overexpression of E-cadherin in EAsPC-1 cells resulted in a slight downregulation of β-catenin levels. The downregulation of β-catenin in response to E-cadherin transduction has been reported by others and it can be explained by the activation of N-cadherin, which further activates AKT (Pece et al., 1999; Fang et al., 2007; De Santis et al., 2009; Zhang et al., 2013). The activation of AKT may not only account for the downregulation of β-catenin, but also for the upregulation of Snail (Xu et al., 2015). The expression of Akt, which is increased in response to the increase in E-cadherin levels, induced Snail expression in both cell lines with a significantly higher induction in EAsPC-1 cells. This higher increase in Snail levels seen in EAsPC-1 cells can be explained by their higher expression of NF-κB, which binds directly to the promoter of Snail and controls its transcriptional upregulation (Pires et al., 2017). Hence, these findings imply that there is a contrast between the expression patterns of EMT-related genes in EAsPC-1 cells and EPanc-1 cells in response to exogenous expression of E-cadherin.

For example, ZEB-1 upregulation is also seen in EPanc-1 cells, but not in EAsPC-1 cells, where we detected rather a decline in ZEB-1 levels in response to the increase in E-cadherin levels. Moreover, the transduction of EPanc-1 cells with E-cadherin yields increases in the expression not only in ZEB-1, but also in Snail, which could imply that a hybrid E/M state prevails. As shown by Kroger et al., hybrid E/M state is a potent enhancer of tumorigenicity (Kroger et al., 2019). They also claim that in the absence of Zeb-1, Snail expression triggers the entry of the cells into the E/M interphase from the E-state, hence Zeb-1 is critical for Snail-induced transition into M state (Kroger et al., 2019).

Upon induction of exogenous E-cadherin expression in EPanc-1 cells that concurrently express Snail and Zeb-1, the absence of them inhibits the transition to M state. We suggest that EPanc-1 cells were positioned into a more epithelial-like (E-like) state due to the increased Zeb-1 and enhanced Snail expressions, whereas EAsPC-1 cells stayed in the E/M hybrid state. 

Met, a tyrosine kinase, phosphorylates the cytoplasmic domain of E-cadherin and promotes its internalization (Aparicio et al., 2012). The functional impact of Met downregulation is reflected in our scratch assay results, where the gap closure was decreased in E-cadherin-overexpressing cells compared to cPanc-1, indicating that the metastatic potential of EPanc-1 cells is hampered. On the other hand, slight upregulation of Met in EAsPC-1 cells suggests that the cells retained their migratory capacity.  

High plasticity is an essential property of cancer cells that disseminate, adapt to the new microenvironment, and survive within a quiescent state. EMT is involved in processes related to both dissemination of tumor cells and their gain of high plasticity. However, when cells stay in a hybrid state where cells present both E and M characteristics rather than E or M alone, their degree of tumorigenicity, as well as plasticity, aggravates (Kroger et al., 2019). In other words, bearing partial EMT characteristics is found in association with the highest degree of plasticity contributing to self-renewal (Weidenfeld and Barkan, 2018; Zhu et al., 2018). Klf4, which is a tumor suppressor for pancreatic cancer, is positively correlated with E-cadherin expression (Zammarchi et al., 2011). Our study confirms the positive correlation between Klf4 and E-cadherin expression. Furthermore, we observed a diminished effect in colony and sphere formation related to increased KLF4 expressionin EPanc-1 cells (Yan et al., 2016). Since EAsPC-1 cells do not express Klf4 to a similar extent and their Nanog expression is significantly increased, EAsPC-1 cells display enhanced sphere-forming capacity. From these findings we conclude that the expression level of Klf4 by itself may not be sufficient to exert a robust effect, rather, Nanog might play a more potentiating role in sphere formation in metastatic pancreatic cancer. To validate this outcome, future studies demonstrating the potentiating role of Nanog are warranted. In conclusion, cancer stem cell markers display cumulative effects and their pattern differs between primary and metastatic pancreatic cancers.

## 5. Conclusion

Hybrid E/M is a process in which cells acquire mesenchymal traits while retaining the epithelial state at the same time. In our study, we aimed to show the differential effects of exogenously induced E-cadherin expression on primary pancreatic cancer cells and compared it to those obtained in a metastatic pancreatic cell line. We clearly observed that depending on the initialE-cadherin levels, Panc-1 cells,which originally had lower levels of E-cadherin than AsPC-1 cells, gained a differing transition state that retained their epithelial-like state and lost their sphere-forming and migratory capacity. On the other hand, AsPC-1 cells that already have high levels of E-cadherin acquired a more hybrid E/M state, as indicated by the decrease in Zeb1 expression and a slight increase in their sphere-forming and migratory capacity. To sum up, we can conclude that E-cadherin is a two-edged sword that pushes cells into differential transition states where cells with low levels of E-cadherin have a tendency to stay in a more epithelial-like state with suppressed tumorigenic characteristics, and the ones with high levels of E-cadherin transition into a more hybrid E/M state and become more tumorigenic. 

## Ethical approval

This article does not contain any studies with animals and human tissues performed by any of the authors. 
